# Supplementary material for: Cryo-EM structures of human magnesium channel MRS2 reveal gating and regulatory mechanisms
Source: Nat Commun. 2023 Nov 8;14:7207. doi: 10.1038/s41467-023-42599-3 (PMC10632456; doi:10.1038/s41467-023-42599-3)
Supplement: Supplementary file 3 — Reporting Summary [file 41467_2023_42599_MOESM3_ESM.pdf]

Corresponding author(s): Doreen Matthies
Last updated by author(s): Oct 6, 2023

Reporting Summary

Nature Portfolio wishes to improve the reproducibility of the work that we publish. This form provides structure for consistency and transparency in reporting. For further information on Nature Portfolio policies, see our Editorial Policies and the Editorial Policy Checklist.

Statistics

For all statistical analyses, confirm that the following items are present in the figure legend, table legend, main text, or Methods section.

n/a	Confirmed	
☒	☐	The exact sample size (*n*) for each experimental group/condition, given as a discrete number and unit of measurement
☒	☐	A statement on whether measurements were taken from distinct samples or whether the same sample was measured repeatedly
☒	☐	The statistical test(s) used AND whether they are one- or two-sided<br>*Only common tests should be described solely by name; describe more complex techniques in the Methods section.*
☒	☐	A description of all covariates tested
☒	☐	A description of any assumptions or corrections, such as tests of normality and adjustment for multiple comparisons
☒	☐	A full description of the statistical parameters including central tendency (e.g. means) or other basic estimates (e.g. regression coefficient) AND variation (e.g. standard deviation) or associated estimates of uncertainty (e.g. confidence intervals)
☒	☐	For null hypothesis testing, the test statistic (e.g. *F*, *t*, *r*) with confidence intervals, effect sizes, degrees of freedom and *P* value noted<br>*Give P values as exact values whenever suitable.*
☒	☐	For Bayesian analysis, information on the choice of priors and Markov chain Monte Carlo settings
☒	☐	For hierarchical and complex designs, identification of the appropriate level for tests and full reporting of outcomes
☒	☐	Estimates of effect sizes (e.g. Cohen's *d*, Pearson's *r*), indicating how they were calculated

Our web collection on statistics for biologists contains articles on many of the points above.

Software and code

Policy information about availability of computer code

Data collection	SerialEM (v.4.0.20)
Data analysis	CryoSPARC (v.3.3.2), CisTEM (v1.0.0), COOT (v.0.9.7), Phenix (v.1.20.1-4487), UCSF Chimera (v.1.16), UCSF ChimeraX (v.1.4)

For manuscripts utilizing custom algorithms or software that are central to the research but not yet described in published literature, software must be made available to editors and reviewers. We strongly encourage code deposition in a community repository (e.g. GitHub). See the Nature Portfolio guidelines for submitting code & software for further information.

Data

Policy information about availability of data

All manuscripts must include a data availability statement. This statement should provide the following information, where applicable:

- Accession codes, unique identifiers, or web links for publicly available datasets
- A description of any restrictions on data availability
- For clinical datasets or third party data, please ensure that the statement adheres to our policy

The data that support this study are available from the corresponding author upon request. Cryo-EM maps have been deposited in the Electron Microscopy Data Bank (EMDB) under accession codes EMD-41624 [https://www.ebi.ac.uk/emdb/EMD-41624] (Cryo-EM structure of the human MRS2 magnesium channel under Mg2+ condition); EMD-41628 [https://www.ebi.ac.uk/emdb/EMD-41628] (Cryo-EM structure of the human MRS2 magnesium channel under Mg2+-free condition); EMD-41629 [https://www.ebi.ac.uk/emdb/EMD-41629] (Cryo-EM structure of the human MRS2 magnesium channel under Mg2+ condition (C1 map)); and

EMD-41630 [https://www.ebi.ac.uk/emdb/EMD-41630] (Cryo-EM structure of the human MRS2 magnesium channel under Mg2+-free condition (C1 map)). The atomic coordinates have been deposited in the Protein Data Bank (PDB) under accession codes PDB-8TUL [https://doi.org/10.2210/pdb8tul/pdb] (Cryo-EM structure of the human MRS2 magnesium channel under Mg2+ condition); and PDB-8TUP [https://doi.org/10.2210/pdb8tup/pdb] (Cryo-EM structure of the human MRS2 magnesium channel under Mg2+-free condition). The source data for Supplementary Fig. 1b-c are provided at the end of the Supplementary Information file.

Research involving human participants, their data, or biological material

Policy information about studies with human participants or human data. See also policy information about sex, gender (identity/presentation), and sexual orientation and race, ethnicity and racism.

Reporting on sex and gender	N/A
Reporting on race, ethnicity, or other socially relevant groupings	N/A
Population characteristics	N/A
Recruitment	N/A
Ethics oversight	N/A

Note that full information on the approval of the study protocol must also be provided in the manuscript.

Field-specific reporting

Please select the one below that is the best fit for your research. If you are not sure, read the appropriate sections before making your selection.

☒ Life sciences ☐ Behavioural & social sciences ☐ Ecological, evolutionary & environmental sciences

For a reference copy of the document with all sections, see nature.com/documents/nr-reporting-summary-flat.pdf

Life sciences study design

All studies must disclose on these points even when the disclosure is negative.

Sample size	For cryo-EM imaging, 3,991 and 9,656 movies were collected for MRS2-Mg2+ and MRS2-EDTA, respectively.
Data exclusions	During image processing, cryo-EM images with bad quality (ice contamination, empty hole, high drift) were removed. Junk particles were sorted by classification and discarded, as they will dampen the resolution of the final map. The detailed data processing were summarized in Methods section and Supplementary Fig. 2. These criteria were established in softwares (CryoSPARC) for cryo-EM image processing.
Replication	The Mg2+-dependent E. coli growth assay with gate-abolished mutants were repeated 3 times. Experimental findings were reliably reproduced.
Randomization	For cryo-EM analysis, subsets of randomly selected particles were subjected to the initial rounds of classification, ab-initio model building and 3D refinement.
Blinding	Blinding is not applicable to single particle analysis.

Reporting for specific materials, systems and methods

We require information from authors about some types of materials, experimental systems and methods used in many studies. Here, indicate whether each material, system or method listed is relevant to your study. If you are not sure if a list item applies to your research, read the appropriate section before selecting a response.

Materials & experimental systems

n/a	Involved in the study	
☒	☐	Antibodies
☐	☒	Eukaryotic cell lines
☒	☐	Palaeontology and archaeology
☒	☐	Animals and other organisms
☒	☐	Clinical data
☒	☐	Dual use research of concern
☒	☐	Plants

Methods

n/a	Involved in the study	
☒	☐	ChIP-seq
☒	☐	Flow cytometry
☒	☐	MRI-based neuroimaging

Eukaryotic cell lines

Policy information about cell lines and Sex and Gender in Research

Cell line source(s)	Expi293F cells (Thermo Fisher Scientific, A14527)
Authentication	No additional authentication was performed by the authors of this study.
Mycoplasma contamination	No mycoplasma contamination was found by the supplying company using mycoplasma qPCR assay.
Commonly misidentified lines (See ICLAC register)	No commonly misidentified line was used

Plants

Seed stocks	N/A
Novel plant genotypes	N/A
Authentication	N/A
